# A novel minimally invasive nonanatomical single‐bundle medial collateral ligament reconstruction technique with a short isometric construct Achilles tendon allograft: A surgical description with clinical and radiological outcomes in multiligament knee injury patients

**DOI:** 10.1002/jeo2.70076

**Published:** 2024-12-15

**Authors:** Mohammadreza Minator Sajjadi, Alireza Mirahmadi, Vahid Alizad, Mohammedhasan Dabis, Ali Saeidi, Mohammad Movahedinia

**Affiliations:** ^1^ Orthopaedic Department Shahid Beheshti University of Medical Sciences Tehran Iran; ^2^ Bone, Joint and Related Tissue Research Center Shahid Beheshti University of Medical Sciences Tehran Iran

**Keywords:** Achilles allograft, medial collateral ligament, minimally invasive, multiligament knee injuries, nonanatomical, reconstruction, short isometric construct, single bundle, single tunnel

## Abstract

**Purpose:**

Multiligament knee injuries (MLKIs) involve various ligaments in the knee. Current double‐bundle anatomical reconstructions of the medial collateral ligament (MCL) increase the level of technical complexity, often resulting in the establishment of numerous bone tunnels and different fixation points with additional hardware. To overcome these limitations, we proposed a novel minimally invasive nonanatomical MCL reconstruction with one tibial tunnel in the metaphysis using Achilles allograft in the MLKI setting.

**Methods:**

In a retrospective study, we enroled 24 patients with MLKIs who underwent a new single‐strand short isometric construct (SIC) nonanatomical MCL reconstruction during 2020 and 2021. We fixed an Achilles allograft in one tunnel in the distal femur on the medial epicondyle and one tunnel in the metaphyseal part of the tibia using interference screws at 2 cm below the joint line between the anatomical insertion of the superficial MCL and the posterior oblique ligament. The patients underwent clinical and radiological assessment at the last follow‐up, 1–2 years after the operation, including valgus stress radiographs, range of motion (ROM), Lysholm and International Knee Documentation Committee (IKDC) scores.

**Results:**

The mean postoperative IKDC score was 77.8 (range, 50.1–86.6). The mean Lysholm score was 84.1 ± 11.9 (range, 96–59). The medial knee widening difference (i.e., mean side‐to‐side difference under valgus stress x‐ray) was measured to be 1.2 mm on average. Only two patients (8%) had ROM limitation of 20° in knee flexion, one of which had surgery failure. Results showed a significant statistical difference between the patients' outcomes according to the number of involved ligaments.

**Conclusions:**

This novel SIC‐like technique with a single tibial metaphyseal tunnel demonstrated satisfactory patient‐reported outcome measures, valgus stress radiographs, ROM and a low rate of knee stiffness and graft failure. While the number of injured ligaments in MLKI patients significantly influenced the outcomes, the results remained acceptable across all patients.

**Level of Evidence:**

Level IV.

**Abbreviations**
ACL: anterior cruciate ligament
dMCL: deep MCL
IKDC: International Knee Documentation Committee
LCL: lateral collateral ligament
MCL: medial collateral ligament
MLKIs: multiligament knee injuries
MRI: magnetic resonance imaging
PCL: posterior cruciate ligament
POL: posterior oblique ligament
PROMs: patient‐reported outcome measures
RCTs: randomized controlled trials
ROM: range of motion
SIC: short isometric construct
sMCL: superficial MCL

## INTRODUCTION

Multiligament knee injuries (MLKIs) encompass a spectrum of injuries involving various knee intra‐ and extracapsular ligaments, among other structures like the menisci, nerves and arteries [7]. The incidence rates of concomitant injuries in MLKIs, including meniscal and cartilage injuries, have been reported at 30.4% and 27.5%, respectively [10]. In the context of MLKI, a thorough evaluation is essential to avoid missed diagnoses, and also, understanding the biomechanics and anatomy of the knee joint, along with appropriate repair and reconstruction techniques, is crucial for managing MLKIs effectively [19, 29]. The medial collateral ligament (MCL) tear plays an essential role in MLKI by contributing to valgus instability, which can increase the strain on other ligaments like the anterior cruciate ligament (ACL) and potentially lead to graft failure in ACL reconstructions [19, 27].

Surgical approaches for knee MCL reconstruction include single‐bundle and double‐bundle techniques, with anatomical reconstructions showing superior outcomes compared to nonanatomical methods [4]. Studies comparing anatomical MCL reconstruction to nonanatomical techniques highlight the importance of restoring both superficial and deep components of the MCL for optimal knee stability [3, 18]. Clinical trials have demonstrated that anatomical MCL reconstruction results in significantly better Lysholm and International Knee Documentation Committee (IKDC) scores compared to nonanatomical repair, emphasizing the importance of anatomical fidelity in surgical outcomes [5, 13]. Therefore, when considering MCL reconstruction, prioritizing anatomical techniques that address both superficial and deep components of the ligament is crucial for achieving optimal knee stability and functional outcomes.

However, anatomic reconstruction of both superficial and deep components of the MCL necessitates significant exposure and numerous drill tunnels, which pose a risk of ligament injury when performed simultaneously with the preparation of bone tunnels for the cruciate ligament. In addition, employing double‐bundle graft structures heightens the technical intricacy, frequently leading to the creation of multiple bone tunnels and various fixation points with extra hardware, potentially increasing the overall size of the final structure [4]. These could be the reasons why studies that performed MCL reconstruction using a single bundle and one tunnel on each side have demonstrated excellent results [11, 15]. The ability to restore stability using a single‐strand short isometric construct (SIC) with a solitary graft is advantageous for surgeons, as the lever arm could also resist some of the rotational forces. The single‐strand SIC technique reduces the risk of tunnel convergence during multiple ligament reconstructions. This is particularly crucial when simultaneously reconstructing the posterior cruciate ligament (PCL) and the MCL [3, 28]. Moreover, current anatomical techniques require tibial side fixation in the diaphyseal part, which does not provide strong biomechanical properties [9]. Also, the use of allograft is often encouraged due to the need for several grafts in MLKI patients [21, 23]. To overcome these limitations, we proposed a novel nonanatomical MCL reconstruction with one tibial tunnel in the metaphysis using Achilles allograft in the MLKI setting.

Nonanatomical MCL reconstruction is not common among knee surgeons because, theoretically, it does not fully restore ligamentous restraints on the posteromedial corner. Therefore, it is questionable whether nonanatomical MCL reconstruction can provide valgus and rotational stability throughout the knee range of motion (ROM). The purpose of this study was to investigate the short‐term patient‐reported outcome measures (PROMs), objective knee stability and radiological results of a new single allograft strand nonanatomical MCL reconstruction technique in a retrospective case series of MLKI patients. This study aims to answer whether the new technique we used for MCL reconstruction in MLKI patients could overcome the limitations of existing anatomical reconstructions without causing the worrying complications of nonanatomical reconstruction. We hypothesize that this novel minimally invasive technique will show acceptable outcomes in MLKI patients.

## MATERIALS AND METHODS

### Study population

In a retrospective study, we enroled patients aged more than 18 years old who underwent a new nonanatomical MCL reconstruction technique in our centre during 2020 and 2021. The study received approval from the Institutional Review Board. All participants provided written informed consent. The ethical committee board also approved using data from our database for research purposes (IR.SBMU.MSP.REC.1402.331).

**Figure 1:**
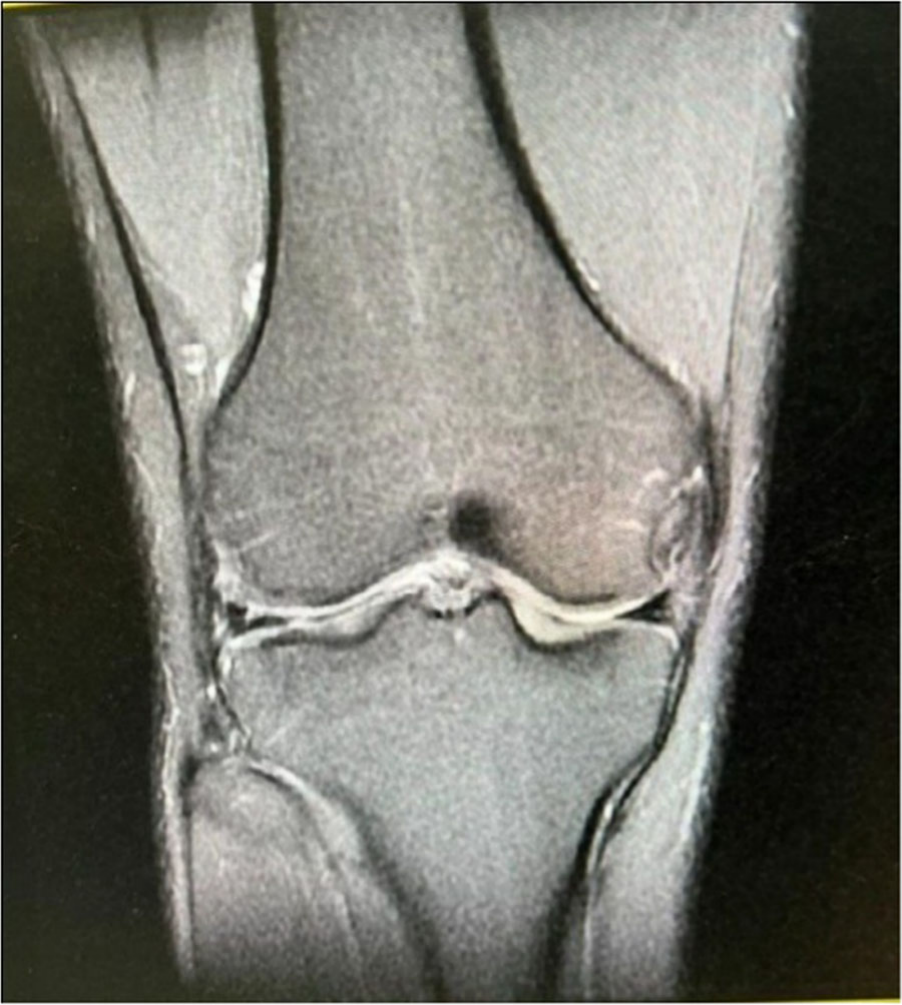
Knee magnetic resonance imaging of patients with mid‐substance medial collateral ligament ligament injury.

Inclusion criteria consisted of patients who were diagnosed with chronic MLKIs (more than 8 weeks) with MCL tear (grade 2 or 3) and underwent one‐stage delayed cruciate and collateral reconstruction. The diagnosis of grade 2 or 3 MCL tear along with meniscus damage or lateral collateral ligament (LCL) and/or cruciate ligament damage was given based on the valgus stress test and magnetic resonance imaging (MRI) (Figure 1). In the valgus stress examination, medial widening of more than 10 mm was considered a grade 3 MCL tear, and between 5 and 10 mm was a grade 2 MCL tear. We excluded MLKIs without MCL tears, patients who had acceptably healed MCL according to clinical and radiological assessments, patients with concomitant around knee fracture and patients who underwent subtotal or total meniscectomy.

Since it has been proved that the number of injured ligaments affects the outcomes of MLKI surgery [22], we also additionally assessed the clinical and radiological outcomes of the new MCL reconstruction technique in two separate groups: patients with two ligament injuries which one of the ligaments were MCL (Group A) and patients with more than two ligament injuries (Group B). This grouping was done to avoid biases of not considering the number of injured ligaments as an influential factor in patients' outcomes [22]. However, as the study's purpose was to introduce a novel technique, we collected data from all patients who underwent surgery with this new technique (considering inclusion and exclusion criteria), and due to the prototype nature of this study and the low number of patients, the study was not powered for this grouping and subsequent analysis.

### Surgical technique

**Figure 2:**
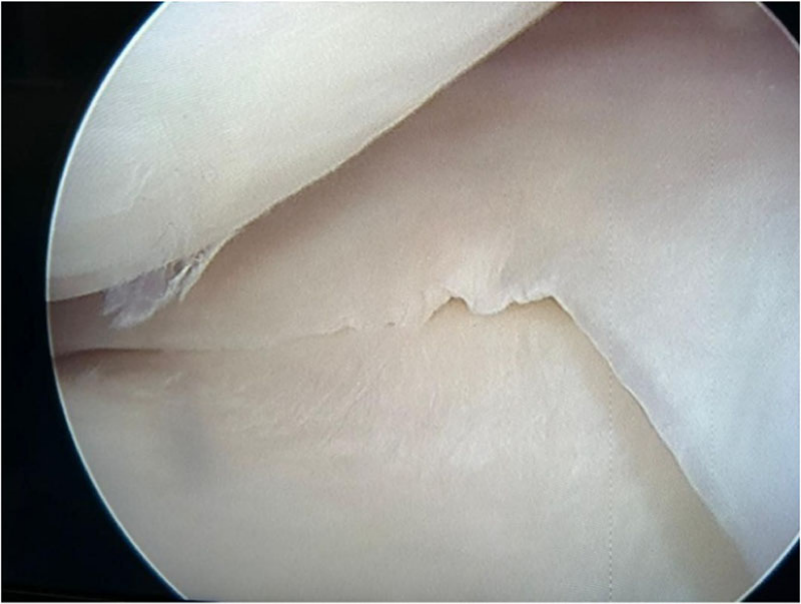
The arthroscopic photograph of the patient shows the medial opening of the knee with valgus stress during the operation.

After induction of general anaesthesia, the patient was placed in the supine position and subjected to an examination using a valgus stress test. The valgus stress test was performed in 20° of knee flexion to estimate the medial gap in the joint line. Then, a diagnostic arthroscopy of the knee was performed to check the intra‐articular injuries. The knee internal anatomy and the medial widening were examined (Figure 2).

Before MCL reconstruction, intra‐articular injuries, including meniscal and cruciate tears, were approached using appropriate techniques for each patient. In cases of concomitant ACL and MCL injuries, the ACL was first reconstructed using a hamstring allograft. The fixation at the femoral end was achieved using an Endobutton through a transportal approach at its anatomical site, and the tibial end was secured with an interference screw at the anatomical location. The PCL reconstruction was performed first using a 9 mm Tibialis posterior allograft for patients with concomitant PCL and MCL injuries. The PCL was fixed at the femoral end with an Endobutton, employing a transportal approach in a single bundle at its anatomical site. At the tibial end, the PCL was fixed through a posteromedial portal using both an interference screw and a cortical screw with a washer at its anatomical location. When addressing a concomitant LCL tear, the LCL was reconstructed following the ACL/PCL reconstruction and before the MCL reconstruction. The LCL and the posterolateral corner were reconstructed using the modified Laprade technique [14], utilizing a 7 mm Achilles allograft (size 21–23 mm).

**Figure 3:**
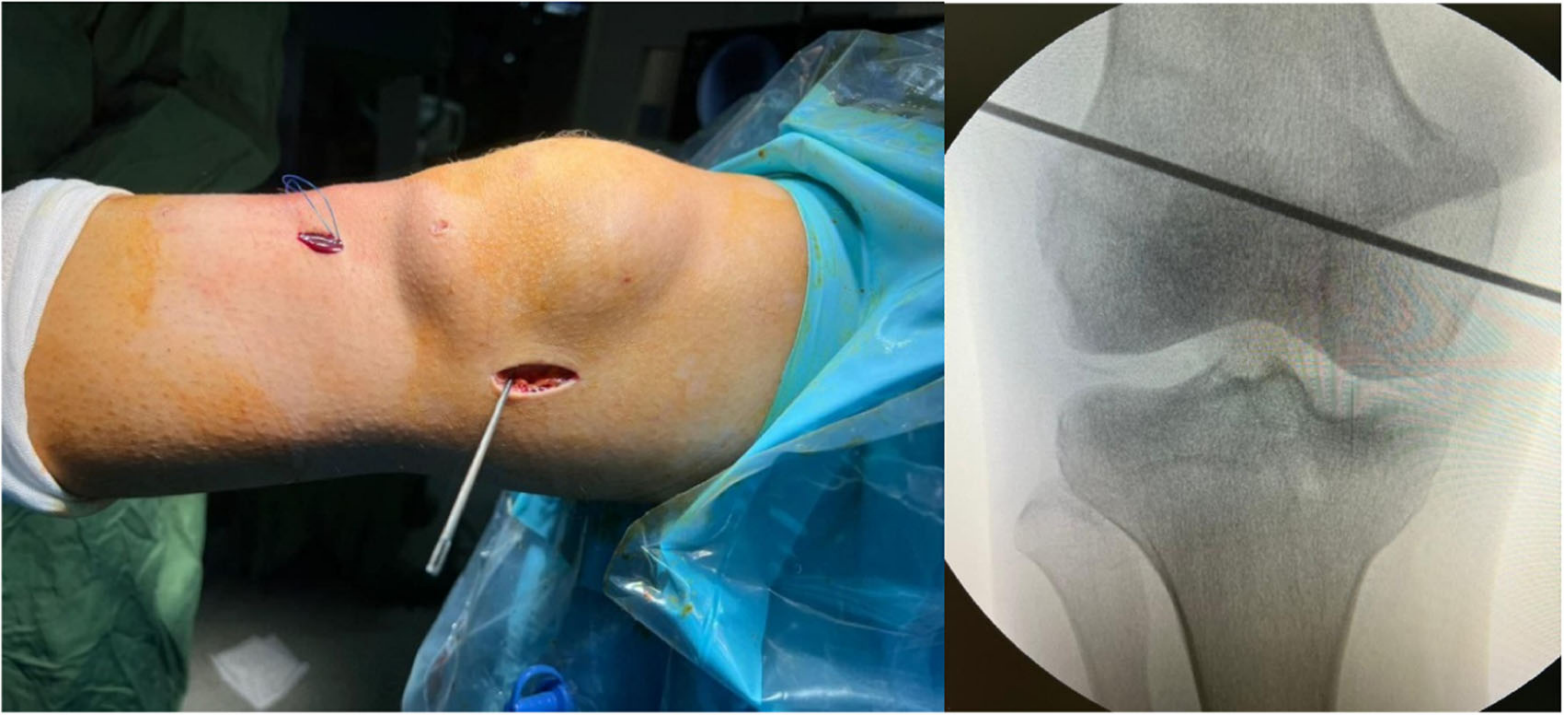
Distal femur approach and guide pin installation for the femoral tunnel.

Regarding the MCL reconstruction, On the femoral side, a 3 cm skin incision was made on the medial epicondyle, and a guide pin was inserted 2–3 mm proximal to the medial epicondyle and 2 mm posterior to it: the anatomical location of the MCL. The guide pin was placed so that it exited from the lateral side. Then, a 35 mm‐long femoral tunnel was created with the help of a reamer (Figure 3).

**Figure 4:**
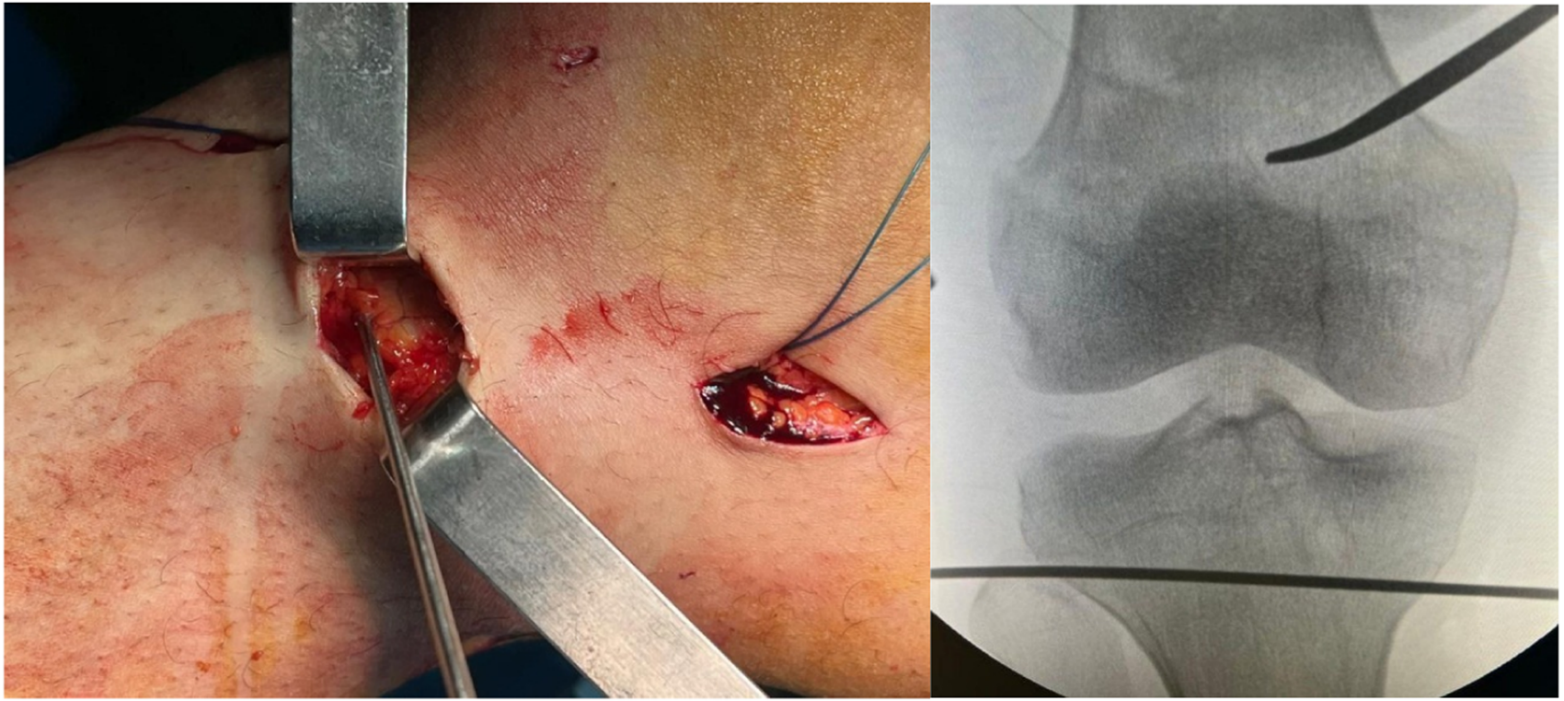
Proximal approach of the tibia to insert a guide pin in the direction of the tibial tunnel.

On the tibial side, a 2 cm skin incision was made on the medial proximal tibia, and a guide pin was inserted 2 cm below the joint line at the metaphyseal part of the tibia and 1 cm anterior to the posteromedial cortex at a point between the anatomical insertion of the superficial MCL (sMCL) and the posterior oblique ligament (POL). The guide pin was positioned to exit from the surface of the Gerdy tubercle and run parallel to the joint line, ensuring protection of the peroneal nerve. Then, a 30 mm‐long tibial tunnel was created with the help of a reamer (Figure 4). Fluoroscopy was utilized to identify the appropriate tibial and femoral tunnels for MCL reconstruction.

**Figure 5:**
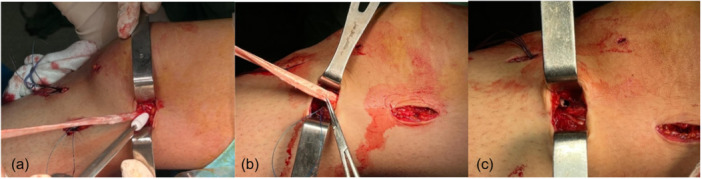
(a) Fixing Achilles allograft by interference screw in femoral tunnel. (b) Passing the Achilles allograft to the tibia tunnel. (c) Fixing the distal part of the allograft in the tibial tunnel with the help of interference screw.

We employed the Achilles allograft to reconstruct the MCL in this new nonanatomical method. During the diagnostic arthroscopy, the assistant prepared the graft sterilely, using a 2–0 Vicryl suture. The prepared Achilles allograft, with a diameter of 8 mm, was fixed within the femoral tunnel using a 9 × 30 mm interference screw. The allograft was then directed into the tibial tunnel, cut to a length 5 mm shorter than the tibial tunnel. The graft was tensioned at the tibial end from the opposite side of the tunnel. As the graft was advanced into the tunnel, an assistant pulled the suture on the other side. Finally, the graft was secured using a 30 × 8 mm interference screw while the knee was positioned in 15–20° of flexion and under varus force (Figure 5).

### Rehabilitation

The patients were given a hinged knee brace, locked in extension. From the 3rd day after the operation, the patient's ROM started with the help of physiotherapy and quadriceps muscle strengthening. Patients were asked to perform isometric strengthening (quadriceps sets and straight leg raises) in the brace and ROM exercises out of the brace for the first 2 weeks postoperatively, intending to reach 90° active ROM. From 2 to 6 weeks, patients continue to work on strengthening exercises and ROM to have full ROM by 6 weeks. If the meniscal repair was not done, the patient was ambulated with partial weight‐bearing the day after the operation for the 1st week, followed by progressive weight‐bearing as tolerated. In the case of meniscal repair, weight‐bearing started after 4 weeks. Muscle strength training was planned based on ACL/PCL reconstruction. We advocated avoiding active isolated hamstring exercises for at least the first 4 months. Patients may be cleared for return to activity after 9 months if they have met strength and stability goals.

### Follow‐up

After discharge, the patients underwent clinical and radiological examinations at 2, 4, 6 and 12 weeks postoperatively. In this study, we presented outcomes of patients' last follow‐up ranging from 1 to 2 years (1.5 years average) after the operation.

**Figure 6:**
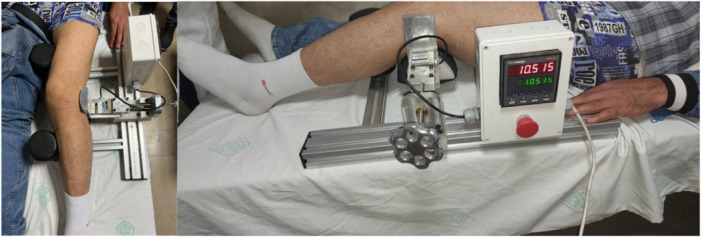
Preparation of valgus stress graph by applying 10 N/M of force at 20° of knee flexion with the help of Bone tech company device after surgery.

Clinical examination in the last follow‐up included knee ROM and valgus stress test. Patients were also assessed using the IKDC and Lysholm scores. Valgus stress radiography was performed using a Telos valgus stress device that applied a force of 10 N/M on the lateral side of the knee in a 20° flexion position [16] (Figure 6). Medial knee widening was assessed for both the healthy and the operated knee (i.e., mean side‐to‐side difference under valgus stress x‐ray).

Although failure criteria are defined differently across previous studies [20], we consider the following criteria for identifying reconstruction failure: Residual medial laxity as a side‐to‐side difference in Rolimeter testing of ≥5 mm or a pivot‐shift grade of ≥2: the need for reoperation due to persistent symptomatic valgus instability; the need for surgical procedures, such as manipulation under anaesthesia for stiffness or surgical debridement for complications; a clinical stress examination demonstrating asymmetric medial gapping (≥2+ laxity) at 30° of flexion; any asymmetric medial gapping observed in full extension; objective IKDC scores classified as C or D; the presence of repair failure or graft rupture confirmed via MRI and the presence of infection.

### Statistical analysis

Statistical analyses were performed using SPSS software version 24 (IBM). Mean ± SD was used to display quantitative variables with normal distribution, and Frequency (%) was used to display qualitative variables. The Kolmogorov–Smirnov test was performed to differentiate parametric and nonparametric data. Radiographic data of valgus stress and Lysholm score had a regular distribution, and a Student's *t* test was used for statistical analysis. IKDC score data had a nonnormal distribution, and the Mann–Whitney statistical test was used for statistical analysis. Qualitative variables were also analyzed using a *χ* ^2^ statistical test. In all analyses, the significance limit of the results was *p* = 0.05.

## RESULTS

The study was conducted on 24 patients (22 men and two women) who underwent a novel nonanatomical MCL reconstruction technique during 2020 and 2021. The time between trauma and surgery was 7.8 months on average (range 2–20 months). All patients with medial knee instability had grades 2 (five patients) and 3 (19 patients). We followed patients for 1–2 years, averaging 1.5 years. The demographic and clinical characteristics of the patients are described in Table 1.

**Table 1:** Demographic and clinical characteristics of the study population.

Characteristics	Mean ± SD or number
Age	32.16 ± 8.12
Gender	
Male	22
Female	2
Associated ligament injury	
ACL‐MCL‐ medial meniscus (Group A)	2
MCL‐ACL (Group A)	14
MCL‐PCL‐medial/lateral meniscus (Group B)	2
MCL‐ACL‐LCL‐ medial meniscus (Group B)	2
MCL‐ACL‐LCL‐lateral meniscus (Group B)	2
MCL‐LCL‐PCL (Group B)	2
Mechanism of injury	
Direct trauma	4
Falling	2
Motor‐car accident	4

Abbreviations: ACL, anterior cruciate ligament; LCL, lateral collateral ligament; MCL, medial collateral ligament; PCL, posterior cruciate ligament.

The patients' mean postoperative IKDC score was 77.8 ± 4.2 (ranging from 50.1 to 86.6). Results showed a significant statistical difference between the patients' outcomes according to the number of involved ligaments, so the IKDC level in group A was significantly higher than in group B (*p* = 0.001) (Table 2). Based on Lysholm scoring, group B patients had good scores, and group A had excellent scores (*p* = 0.011). The mean Lysholm of all patients was 84.1 ± 5.7 (ranging from 59 to 96) (Table 2).

**Table 2:** Radiological valgus opening and functional outcomes.

Variable	Total (mean ± SD)	Group A (mean ± SD)	Group B (mean ± SD)	*p* Value
Medial widening difference (mm)	1.21 ± 0.6	1.06 ± 0.76	1.9 ± 0.28	0.018
IKDC Score	77.82 ± 4.2	84.02 ± 3.5	59.23 ± 7.9	0.001
Lysholm Score	84.16 ± 5.7	90.1 ± 5.1	66.3 ± 6.42	0.011

Abbreviation: IKDC, International Knee Documentation Committee.

The evaluation of stress radiography revealed 1.2 ± 0.6 mm as the mean difference between medial widening of the reconstructed and normal knee with a min of 0.5 mm and max of 2.1 mm. This parameter was statistically higher in group B patients (*p* < 0.01) (Table 2).

Examination of ROM in the studied patients after surgery showed that only two patients in group B had a limitation of 20° in knee flexion. In other patients, ROM was complete, and no extension deficit was found in patients. Regarding the complications, it should also be mentioned that no infection or thromboembolism was seen in any of the studied patients after surgery.

Only one person in group B had a failure in the femoral side of the reconstruction, which was reoperated. This patient was one of the participants with knee flexion limitation. The postoperation medial widening evaluation of this patient was more than 10 mm, and the femoral interference screw was not completely fixed in the femoral tunnel. Two weeks later, revision surgery for this patient was done with the same graft but a larger femoral screw.

## DISCUSSION

The most important finding of the present study was that the new technique of MCL reconstruction presented in this study, in which a single tibial tunnel was inserted in a nonanatomical location, showed acceptable medial stability based on medial widening difference and PROMs measured with IKDC and Lysholm questionnaires. Furthermore, the results demonstrated that this new technique in patients with two ligament injuries yielded a better outcome than in patients with more than two injured ligaments.

Knee stiffness or arthrofibrosis is one of the important complications following surgery in MLKI patients, and it was shown to occur in 11.2% of MLKI patients [10]. Based on previous studies, details related to surgical techniques, such as choosing Achilles allografts, shortening the surgery time and minimizing the soft tissue damage, might prevent ROM limitation [6, 17]. A major concern of knee surgeons that prevents them from using nonanatomical reconstruction methods is the issue of anisometry, which is defined as the possibility of limited ROM due to the location of the ligament in a place other than the anatomical location. Some studies reported that clinical and objective outcomes of anatomic reconstructions were superior to those with nonanatomic reconstruction [4]. However, in our study, the ROM after surgery was full in 91.7% of participants, and in only two patients (8.3%), a limitation was observed in 20° flexion, and these two patients were among the group with multiple ligament involvement, which was aligned with previous studies [22]. The minimally invasive nature of this new technique and the use of an Achilles allograft yielded a good result regarding ROM and knee stiffness.

In various studies that reconstructed the sMCL and POL ligaments by anatomical method, the results of the postoperative PROMs and difference in medial knee widening were similar to our study. The IKDC score reported by Tapasvi et al., who employed the anatomical reconstruction technique, was slightly higher than in our study, with scores increasing from 58 preoperatively to 78.2 postoperatively. The observed average difference in knee widening was also reported as 1.2 mm, aligning with our study's results [25]. It is important to note that Tapasvi et al. studied 34 patients, some of whom had isolated MCL involvement. In contrast, our study focused on MCL reconstruction in patients with two or more ligament tears. When specifically considering patients with only two ligament injuries, our study recorded a higher IKDC score (84.02) compared to Tapasvi's (78.2). Furthermore, Laprade et al. reported that their IKDC scores improved from 43.5 to 76.2 following anatomical MCL reconstruction [12]. While our study does not provide preoperative scores, the final IKDC score in our study surpassed that of Laprade's. Additionally, our findings showed a more favourable average difference in medial knee widening compared to Laprade's (1.2 vs. 1.3 mm) [12]. This new technique effectively reduced the average difference in medial knee widening among the patients in this study compared to the accepted difference between injured and noninjured MCL reported in previous studies (1.2 vs. 2.3 mm) [16].

On the other hand, studies that utilized a single tibial tunnel for sMCL reconstruction reported outcomes that were even more favourable than ours. For instance, the study by Kitamura et al. revealed an improvement in the PROMs as the overall Lysholm score averaged 94.8, which surpasses our research result [11]. Additionally, they demonstrated that the side‐to‐side difference was <3 mm in 86.7% of the patients, with an average difference of 0.3 mm observed in patients who underwent MCL/ACL/PCL reconstruction, again indicating better outcomes than ours. Similarly, the postoperative Lysholm scores were 88.6 in Liu et al.'s study [15], which is more favourable than our findings. The superior results in these studies compared to MCL reconstruction involving two tibial tunnels may suggest that double‐bundle anatomical MCL reconstruction requires extensive exposure and multiple drill tunnels, potentially impacting outcomes negatively. Moreover, the better results in these referenced studies compared to our study may be attributed to a lower proportion of patients with multiple ligament injuries in these studies. Our study included a higher percentage of this group of patients, with 33.3% of patients sustaining more than two ligament injuries.

**Figure 7:**
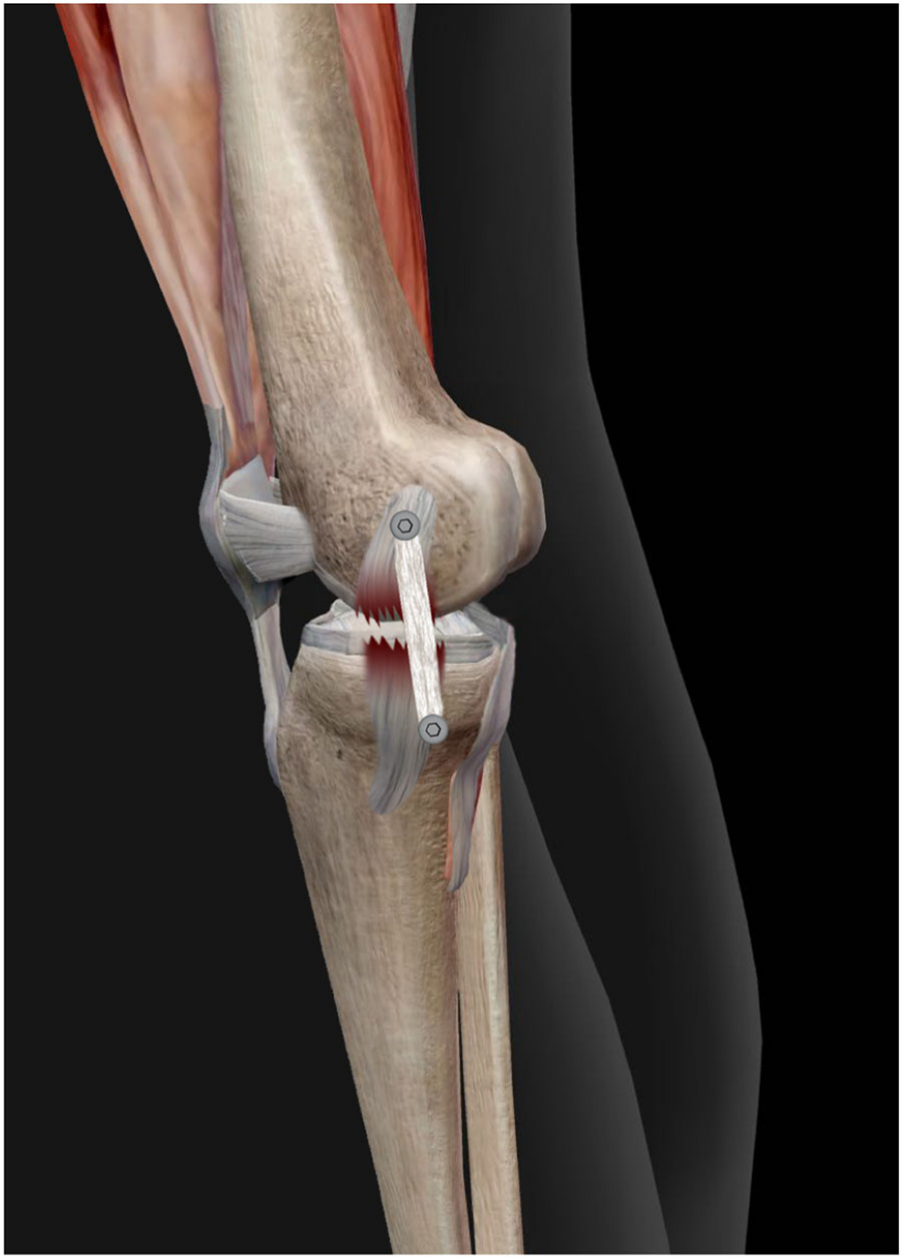
Schematic view of the tibial and femoral tunnel location of this new nonanatomical technique.

The acceptable results of our study can be attributed to several advantages that can be considered for our new nonanatomical MCL reconstruction, such as a minimal approach, reducing the surgical time, using Achilles allograft, simplifying the surgical technique and reducing the number of tibial and femoral tunnels created for reconstruction. On the other hand, because the tibial tunnel of the MCL was inserted at the proximal level than the sMCL attachment, fixation with interference screws was done in the metaphyseal part of the tibia, which creates more strength in the reconstructed structure in comparison to the diaphysis (Figure 7). Also, the logic of using an SIC reconstruction increases this technique's effectiveness compared to other nonanatomical MCL reconstructions.

**Figure 8:**
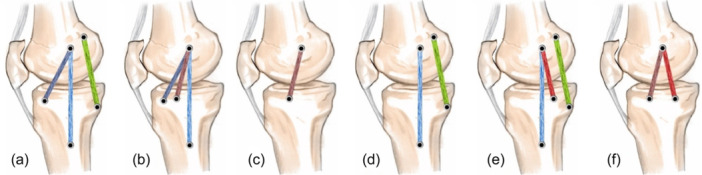
Different techniques in medial collateral ligament (MCL) reconstruction. Purple Bundle: anatomic deep MCL (dMCL), Blue Bundle: anatomic superficial MCL (sMCL), Green Bundle: anatomic posterior oblique ligament (POL), Brown Bundle: nonanatomic short isometric construct (SIC) Bundle (reported by Borque et al.), Red Bundle: Our nonanatomic SIC‐like bundle. (a) MCL reconstruction with anatomical reconstructing of sMCL, dMCL and POL bundles, (b) MCL reconstruction with anatomical reconstructing of dMCL and sMCL and nonanatomical reconstructing of SIC bundle, (c) MCL reconstruction with single tunnel SIC bundle, (d) MCL reconstruction with anatomic sMCL and POL (Laprade technique), (e) MCL reconstruction with Laprade technique + Our introduce Bundle, (f) comparing Borque et al. SIC technique with ours.

As previous studies showed, SIC was founded on new anatomical evidence indicating that the fibres of the medial ligament complex, which insert directly into the medial epicondyle of the femur, are the only isometric fibres [26]. This hypothesis led to the development of a new technique by Borque et al. that utilizes an isometric graft fixed on the medial epicondyle of the femur and the tibia at a point 20 mm below the articular margin at the halfway point between the anterior and posterior margins of the sMCL [2]. Studies demonstrated that single tunnel SIC shows an acceptable result regarding anterior translation, external rotation, varus rotation and valgus instability [3, 24]. These studies established that positioning the MCL tibial insertion between the sMCL and deep MCL (dMCL) anatomical sites yields acceptable results. In the current study, we applied this concept in our novel technique by selecting the MCL tibial insertion position to uphold the SIC concept. However, previous SIC studies overlooked the function of the POL. To enhance reconstruction outcomes, we positioned the MCL tibial insertion between the POL and sMCL anatomical sites, aiming to restore the functions of both the sMCL and dMCL, as well as the POL (Figure 8). By placing the tibial tunnel between the sMCL and POL anatomical insertion sites, we theoretically restored the biomechanics of both MCL (sMCL and dMCL) and POL structures with a single tunnel. We clinically evaluate the efficiency of this technique, but further biomechanical studies are required to confirm these findings and to confirm the SIC nature of our new technique.

MCL injuries can occur in various elective and traumatic conditions, and factors influencing treatment outcomes have been demonstrated in previous studies [1, 21]. As mentioned, more ligament injuries during MLKI affect the clinical outcomes of the surgery [22]. During the study, we found a significant relationship between the number of ligament injuries and the outcomes of the patients. For this reason, we categorized the patients into two separate groups: group A, patients with two ligament injuries in which one of the ligaments was MCL, and Group B (>2 ligaments), who had torn MCL tears with two other ligaments. It should be noted that none of the patients in our study had isolated MCL injury. Although it was not the main hypothesis of this study, we found that all the indicators examined in this study (valgus stress, ROM, Lysholm, IKDC) showed a significant improvement in patients with less ligament damage compared to patients with more ligament involvement using our new technique. This finding aligns with previous studies that indicate that more severe injuries affecting multiple ligaments that necessitate surgical intervention on three or more ligaments are associated with an increased risk of stiffness and lower PORMs following MLKI surgery [8, 22].

This study introduced a prototype study of a novel minimally invasive MCL reconstruction technique in MLKI sitting. In this technique, a single metaphyseal tibial tunnel was inserted in a nonanatomic location based on the SIC concept, and an Achilles allograft was used to reconstruct the patient's MCL. This technique showed acceptable results in clinical and radiological evaluations.

This study had several limitations, including the retrospective nature of the study, which was the reason for the lack of preoperative assessments. Also, we do not have a comparative group with other MCL reconstruction or repair techniques. However, in the discussion, we compare our results with those of previous studies on other MCL reconstruction techniques. Furthermore, this study did not evaluate long‐term outcomes for 5 years or more. Due to the low possibility of access to patients with isolated MCL injury, we studied patients with MLKIs. The presence of different injuries other than isolated MCL tears may have affected the results. Due to the diversity of injuries in MLKIs and our low participant population, the distribution of patients among different combinations of injuries was not normally distributed and did not have high power to conclude a definite claim based on the results. This issue could have affected the results. For example, there is a technical challenge regarding the possibility of tibial tunnels interfering in cases with concomitant MCL and PCL tears. Although we lowered this challenge by reducing the length of the tibial tunnel to 30 mm, the low number of patients with MCL and PCL injuries (four patients) may have influenced the overall results. Future randomized controlled trials (RCTs) with proper sample size are required to confirm the findings of this study.

## CONCLUSION

This novel minimally invasive short construct technique used an Achilles allograft with a single tibial tunnel and demonstrated satisfactory results regarding PROMs, valgus stress radiographs, ROM and the incidence of knee stiffness. A low failure rate was reported with this technique. While the number of injured ligaments in MLKI patients significantly influenced the outcomes, the results remained acceptable across all patients. Future RCTs with proper sample size are required to confirm the findings of this study.

## AUTHOR CONTRIBUTIONS

Mohammadreza Minator Sajjadi designed the study and proposed the new technique used in this study and was the main surgeon of the patients. He carried out the postoperative visits and has given final approval for the version to be published. Alireza Mirahmadi, Vahid Alizad and Ali Saeidi participated in the study drafting and design. Alireza Mirahmadi and Ali Saeidi helped Mohammadreza Minator Sajjadi during surgery and postoperative visits and participated in data gathering. Mohammedhasan Dabis carried out data analysis and wrote the first draft. Mohammad Movahedinia contributed to the first draft of the manuscript, and Alireza Mirahmadi revised it critically for important intellectual content. All authors read and approved the final manuscript.

## CONFLICT OF INTEREST STATEMENT

The authors declare no conflict of interest.

## ETHICS STATEMENT

The study received approval from the Institutional Review Board (IR.SBMU.MSP.REC.1402.331). Informed consent was obtained from all the participants.

## Data Availability

The data sets generated and/or analyzed during the current study are not publicly available due to the rules of our institute but are available from the corresponding author on reasonable request.
